# A phase 1, single-center, randomized, dose finding study to evaluate the safety, tolerability, and immunogenicity of a prophylactic COVID-19 pDNA vaccine after single ascending doses in healthy adults

**DOI:** 10.3389/fmed.2026.1781420

**Published:** 2026-04-15

**Authors:** Iman Almansour-Alzamil

**Affiliations:** 1 Nucleic Acid Vaccine Lab (NAVL), Department of Epidemic Diseases Research, Institute for Research and Medical Consultations (IRMC), Imam Abdulrahman Bin Faisal University, Dammam, Saudi Arabia; 2 Clinical Biomanufacturing Facility (CBMF), Institute for Research and Medical Consultations (IRMC), Imam Abdulrahman Bin Faisal University, Dammam, Saudi Arabia; 3 Department of Genetic Research, Institute for Research and Medical Consultations (IRMC), Imam Abdulrahman Bin Faisal University, Dammam, Saudi Arabia

**Keywords:** booster, COVID-19, heterologous, mRNA, pDNA, SARS-CoV-2, vaccine

## Abstract

**Background:**

SARS-CoV-2 has been responsible for a worldwide public health crisis and a pandemic that began in February 2020, with approximately 679 million cases and over 6.7 million deaths to date. Despite the existence of approved COVID-19 vaccines, these vaccines are either based on mRNA technology or viral vectors. Imam Abdulrahman Bin Faisal University (IAU) has developed a thermally stable plasmid DNA (pDNA)-based vaccine candidate using a platform approach that enables the rapid development of vaccines against emerging viral diseases. The pDNA vaccine developed by IAU encodes the full-length, optimized version of the SARS-CoV-2 Spike protein. Key advantages of this pDNA vaccine are that it is cost-effective, thermally stable, and can be used to administer multiple immunizations without incurring anti-vector responses, making it a promising candidate as a vaccine booster dose.

**Methods:**

This study aims to investigate the safety, tolerability, and immunogenicity of this prophylactic pDNA vaccine as a booster vaccine to protect against COVID-19 when administered as an intramuscular injection in a single ascending dose design. In total, 42 eligible healthy subjects will be enrolled in the study. The subjects will be randomized to receive either the investigational vaccine or placebo (Saline) at a 3:1 allocation ratio. Participants will have received two doses of a SARS-CoV-2 mRNA vaccine at least 4 months prior to enrollment. Subjects will be enrolled in a dose-escalating study and assigned to one of the three cohorts (Cohort 1: low-dose; Cohort 2: mid-dose; Cohort 3: high-dose). Primary endpoints will include local reactions and systemic reactions for up to 7 days following vaccination, adverse events from vaccination to 1 month, and serious adverse events from vaccination to 6 months. Secondary endpoints will include the evaluation of binding antibody (bAB) and neutralizing antibody (nAB) responses at vaccination day (Visits 1) and 30 days after vaccination (Visit 4), the ratio of bAB to nAB titers at Visits 1 and 4, and cellular immune responses (CD3, CD4, CD8, CD45RA, CCR7, IFN-γ, IL-2, IL-4, IL-13, and TNF) at Visits 1 and 4.

**Discussion:**

The study will be critical to determine the safety and immunogenicity of the pDNA vaccine in participants who have previously received a COVID-19 mRNA vaccine. This study will also aim to determine the safest maximum tolerated dose in participants receiving the pDNA vaccine.

**Trial registration:**

ClinicalTrials.gov SCTR No 23010802.

## Introduction

### Background and rationale {6a}

The rapid development of vaccines against Severe Acute Respiratory Syndrome Coronavirus 2 (SARS-CoV-2) was a significant scientific achievement in modern history, with multiple vaccines receiving authorization and approval for emergency use (1–4). Despite these efforts, the burden of COVID-19 remains substantial, particularly in low to middle income countries (LMIC), where lower vaccination rate contributes to the contious emergence of variants of concern (VOC) (5). Besides, vaccine side effects have been documented, including the risk of myocarditis and pericarditis in adolescents and young adults aged 18–24 (4, 6, 7). Furthermore, individuals with a history of allergic reactions may have an elevated risk of an allergic reaction following mRNA vaccination, likely due to the PEG component included in the lipid nanoparticles of mRNA vaccines components (8). Given that the current mRNA vaccines strictly require ultra-cold storage to remain effective, there is an ongoing need for developing thermally stable vaccines that can be easily distributed and stored, especially in countries lacking robust ultra clod chain infrastructure (9, 10).

For these reasons, there is a clear need to explore alternative vaccine platforms that can overcome the limitations associated with currently approved COVID-19 vaccines. Plasmid DNA (pDNA) vaccine technology has an inherit ability to produce a targe antigen which then trigger immune responses (11–13).

Recent years have seen rapid improvements in the development of pDNA vaccine platforms, with various pDNA vaccine candidates having progressed to Phase II/III clinical trials (14–19). There are several advantages to pDNA vaccines. For one, when compared with mRNA vaccine platforms, pDNA is more stable, such that ultra cold-chain shipment and storage are not needed (11, 12). In addition, the potential for anti-vector immunity generated after immunization with a viral vector vaccine is not a concern for the pDNA vaccine platform. Moreover, pDNA vaccine platform is generic and adaptable to high scale manufacturing (11, 12). While concerns regarding the theoretical potential for vector DNA integration have been raised, previous preclinical and clinical studies on the tested DNA vaccine candidates following intramuscular immunization revealed no evidence of such integration into the genomes of host cells (20, 21). However, one notable disadvantage of the pDNA vaccine platform is that it tends to be associated with relatively low immunogenicity in humans. This finding has spurred a range of approaches to enhancing immunogenicity, such as gene insert optimization (12).

Imam Abdulrahman Bin Faisal University (IAU) has developed an investigational prophylactic COVID-19 pDNA vaccine using a codon-optimized version of the SARS-CoV-2 gene encoding the spike (S) protein (S.opt.FL). This vaccine (Almansour-001), consists of a pDNA carrying an optimized, full lenghth Spike (S.opt.FL) gene insert (22) from the wild-type SARS-CoV-2. The plasmid backbone included in the study is (pVAX-1), an FDA-approved plasmid for application in clinical trials. Moreover, the vaccine is administered intramuscularly (IM) without the need of physical delivery approaches, such as electroporation or gene gun. The preclinical study conducted at IAU has demonstrated that S.opt.FL is immunogenic in mice (22). The study found that S.opt.FL elicited high binding antibody (bAB) and neutralizing antibody (nAB) as well as a strong interferon (IFN)-γ response consistent with cellular immunity (22). In addition, a subsequent protective efficacy study in Syrina Golden hemesters indicated that this S.opt.FL pDNA vaccine candidate is safe and effective in preventing SARS-CoV-2 infection (23).

Previous work on pDNA vaccines encoding the S gene of SARS-CoV, MERS-CoV, and SARS-CoV-2 has demonstrated that pDNA-based vaccines are safe and well-tolerated (24–26). Due to increasing vaccination coverage worldwide, the WHO and International Coalition of Medicine and Regulatory Authority (ICMRA) have issued guidance regarding the design of clinical trials for next-generation COVID-19 vaccines in the context of the availability of authorized COVID-19 vaccines. These recommendations include the testing of next-generation vaccines as booster doses in vaccinated individuals (27).

The purpose of this clinical trial is to evaluate the safety and immunogenicity of a single dose of an investigational pDNA vaccine encoding the S.opt.FL gene of inserted into the pVAX1 plasmid relative to placebo control. In this Phase 1 study, the pDNA vaccine candidate will be administered intramuscularly (IM) as a booster dose to immunize healthy adults previously vaccinated with mRNA vaccine (18–55 years). The utilized S.opt.FL pDNA vaccine will be evaluated through a single ascending dose study design with three different cohorts administered low, intermediate, and high doses of pDNA.

#### Dose rationale

The starting dose selected for this study was based on the starting dose of 1 mg/ml used in the pivotal non-clinical toxicity study. The repeated toxicity study was designed in line with the immunization schedule in this study, reflected by the number of doses and immunization intervals. Details are described in the Investigator's Brochure (IB). The tested S.opt.FL COVID-19 pDNA vaccine candidate (Almansour-001) demonstrated high immunogenicity and a good safety profile in multiple tested animals (Mice, Syrian Golden Hamsters, New Zealand White Rabbits). Further, the repeated dose toxicology study demonstrated the safety and tolerability of the clinical-grade preparation of the investigational vaccine. No vaccine-related clinical signs or mortality were observed.

#### Risk-benefit assessment

##### Risk and mitigation

The potential risks associated with study participation will be minimized by implementing adequate inclusion/exclusion criteria. Risks associated with any vaccination include local injection site reactions (i.e., pain, redness, and swelling at the injection site) and systemic events (including fever, fatigue, headache, chills, vomiting, diarrhea, muscle and joint pain) which will be carefully monitored through safety evaluation procedures. Potential risks that may be associated with the COVID-19 pDNA vaccine product are summarized in Table 1.

**Table 1:** Risk-benefit assessment.

Potential risk	Data/Rationale of the risk	Mitigation strategy
Potential risk of local reaction (pain, redness, and swelling at the injection site) and systemic events (fever, fatigue, headache, chills, vomiting, diarrhea, muscle and joint pain)	These potential adverse reactions are also associated with other vaccines and are described in the protocol through the implementation of FDA guidance for toxicity grading	The study protocol design includes the implementation of controlled vaccination per cohort that is closely monitored to limit the rate of enrollment to ensure participant safety. The first two participants for each randomized cohort will be observed for 4 h to assess any immediate AEs, with each subject being monitored for 60 min before proceeding to the next subject. The next four participants will be dosed only after 48 h and after safety review of the first four participants, and each will be monitored for 2 h. The rest of the subjects (*n* = 10) will be enrolled after 24 h and will be monitored for 2 h. Dose escalation for the subjects in the next cohort will not occur until 12–14 days and after DMC reviews the accumulated safety data. Stopping rules are also applied in the protocol. The study employs the use of a diary to monitor local reactions and systemic reactions. Safety measures are instituted to closely monitor participants' safety during this clinical study and to assess risks associated with administration of the investigational product.
Potential unknown AEs and laboratory abnormalities associated with novel vaccine	This is a first–in–human Phase I study with clinical data available	The study protocol design includes the implementation of controlled vaccination per cohort that is closely monitored to limit the rate of enrollment to ensure participant safety. The first two participants for each randomized cohort will be observed for 4 h to assess any immediate AEs, with each subject being monitored for 60 min before proceeding to the next subject. The next four participants will be dosed only after 48 h and after safety review of the first four participants, and each will be monitored for 2 h. The rest of the subjects (*n* = 10) will be enrolled after 24 h and will be monitored for 2 h. Dose escalation for the subjects in the next cohort will not occur until 12–14 days and after DMC reviews the accumulated safety data. Stopping rules are also applied in the protocol. The study employs the use of a diary to monitor local reactions and systemic reactions. Safety measures are instituted to closely monitor participants' safety during this clinical study and to assess risks associated with administration of the investigational product.
Theoretical risk of possible DNA integration and prolonged expression	The risk that a pDNA vaccine may integrate into the recipient's DNA has been only theoretical to date	The plasmid backbone of IAU COVID-19 vaccine is an FDA-approved plasmid (pVAX1) that has been tested in numerous clinical trials with an excellent safety profile. The pVAX1 plasmid does not encode a mammalian expression system or molecular adjuvants.
Low immunogenicity	Low pDNA vaccine–associated immunogenicity in humans	Codon optimizations of the S gene insert was implemented to increase expression.

##### Benefits

There are no known direct benefits to the participants enrolled in this study. The IAU COVID-19 pDNA vaccine is expected to elicit an immune response against the original SARS-CoV-2 strain. Information gained from this trial will help with the following:

- To support the ongoing development of an improved COVID-19 vaccine that can overcome the limitations of currently approved vaccines.
- To support the clinical development and application of pDNA vaccine platforms.

##### Overall risk-benefit assessment

The IAU COVID-19 vaccine is in the early clinical stages of testing, as it has not undergone prior clinical testing. However, considering the minimal potential risks associated with the study and the medical measures that will be implemented to minimize the risk to healthy subjects, the overall risks are justified by the potential benefit linked to the development of an improved COVID-19 vaccine.

### Objectives {7}

The objective of this study is to evaluate the safety, tolerability, and immunogenicity of the COVID-19 pDNA vaccine product in healthy individuals through a dose escalation study with a single ascending dose design. The trial objectives, assessment, and endpoints are summarized in Table 2.

**Table 2:** Trial objectives, estimands, and endpoints of the phase-I clinical trial.

Objectives	Estimands	Endpoints
Primary	Primary	Primary
To assess the safety and tolerability of the investigational COVID-19 pDNA vaccine compared to placebo	• Local reactions for up to 7 days following Vaccination (Visit 1) • Systemic events for up to 7 days following Vaccination (Visit 1) • AEs from vaccination (Visit 1) to (Visit 4) • SAEs from vaccination (Visit 1) to (Visit 5)	• Assessment of administration site reactions (local reactions) • Assessment of systemic AEs • Clinical assessment of participants health status • Clinical laboratory investigations • Monitoring of participants' COVID-19 infection status • Recording of solicited and unsolicited AEs and SAEs
Secondary	Secondary	Secondary
To assess binding and neutralizing antibody responses to the investigational COVID-19 pDNA vaccine compared to placebo	• bAB responses at (Visit 1) and (Visit 4) • nAB responses to the investigational pDNA vaccine at (Visit 1) and (Visit 4) • Ratio of bAB and nAB titers at (Visit 1) and (Visit 4). • Fold rise (GMFR) in serum anti-S, anti-S1, and anti-RBD binding antibody titers at (Visit 1) and (Visit 4).	• bAb responses against the S, S1, and RBD proteins of SARS-CoV-2 • Fold rise in bAB responses against the S, S1, and RBD proteins of SARS-CoV-2 • nAb responses against the S protein of SARS-CoV-2 • Fold rise in anti-S nAB titers for SARS-CoV-2 • bAB and nAB ratios for the S protein of SARS-CoV-2
To assess T cell and intracellular cytokine responses to the investigational COVID-19 pDNA vaccine compared to placebo	• Cellular immune response at (Visit 1) and (Visit 4)	Cellular immune responses (CD3, CD4, CD8, CD45RA, CCR IFN-γ, IL-2, IL-4, IL-13, and TNF)
Exploratory Objective	Exploratory	Exploratory
Humoral Response to SARS-CoV-2 Variants of Concern in response to the investigational COVID-19 pDNA vaccine compared to placebo	• bAB responses against SARS-CoV-2 VoC • nAB responses against SARS-CoV-2 VoC • bAB and nAB ratio for SARS-CoV-2 VoC	• bAB responses against SARS-CoV-2 VoC): (alpha, beta, Gamma, Delta, and Omicron) • nAB responses against SARS-CoV-2 VoC): (alpha, beta, Gamma, Delta, and Omicron) • bAB and nAB ratio for SARS-CoV-2 VoC): (alpha, beta, Gamma, Delta, and Omicron)

AEs, adverse events; SAE, severe adverse events; bAB, binding antibody; nAB, neutralizing antibody.

#### Primary objective (safety and tolerability)

- To assess the safety and tolerability of the investigational pDNA COVID-19 vaccine in healthy adults when administered as a single dose vs. placebo.

##### Assessments

- Local reactions (i.e., reactogenicity) for up to 7 days following vaccination.
- Systemic events for up to 7 days following vaccination.
- Adverse events (AEs) for up to 1 month following vaccination.
- Serious AEs (SAEs) for up to 6 months following vaccination.

#### Secondary objectives (immunogenicity)

- To assess S-specific bAB and nAB responses as indicators of humoral immune response after vaccination compared to placebo.
- To assess T cell and intracellular cytokine responses as indicators of cell-mediated immune response after vaccination compared to placebo.

##### Assessments: humoral response to wild-type SARS-CoV-2

- Evaluation of S-specific bAB responses to the investigational pDNA vaccine vs. placebo at Visit 1 (baseline) and Visit 4 (30 days after Visit 1).
- Evaluation of S-specific nAB responses to the investigational pDNA vaccine vs. placebo at Visit 1 and Visit 4.
- Comparison of the ratio of bAB to nAB titers directed against the investigational pDNA vaccine vs. placebo at Visit 1 and Visit 4.
- Determination of the geometric mean fold rise (GMFR) in the serum S-specific, anti-S1-specific, and anti-RBD bAB titers directed against the investigational pDNA vaccine vs. placebo at Visit 1 and Visit 4.

##### Assessments: cell-mediated Responses to wild-type SARS-CoV-2

- Evaluation of cellular immune response by measuring CD3, CD4, CD8, CD45RA, CCR7, IFN-γ, IL-2, IL-4, IL-13, and TNF after investigational pDNA vaccine vs. placebo.

#### Exploratory objective (immunogenicity)

- To further explore the immunogenicity of the investigational pDNA vaccine against the SARS-CoV-2 variants of concern in vaccinated subjects vs. placebo.

### Trial Design {8}

This investigational vaccine study is a Phase 1, randomized, dose-finding, single-center study, enrolling healthy adults. In total, 42 healthy individuals will be enrolled in the study. The eligible individuals will be randomized to receive either the investigational vaccine or placebo (Saline). Subjects will be enrolled at a 1:1:1 ratio in a dose-escalating manner to one of three cohorts (Cohort 1: low dose level, “1 mg”; Cohort 2: mid-dose level, “2 mg”; Cohort 3: high-dose level, “4 mg”). All subjects will receive a single dose of the vaccine or placebo IM.

The study duration for an individual subject is approximately 7 months. Interim analysis for safety will be performed on the data that are collected from all study subjects at Visit 1, Visit 2 (1 day after Visit 1), Visit 3 (7 days after Visit 1), and Visit 4, while interim analysis for immunogenicity will be performed on the data that are collected from all study subjects at Visit 1 and Visit 4. Table 3 illustrates the schedule of activities (SOA) and timing of study visits and assessments.

**Table 3:** Schedule of study visits and assessments—Visit 1 to Visit 5.

Visit number	Screening	1	2	3	4	5	Unplanned	Unplanned
Visit description	Screening	Vax 1	Next-Day Follow-up Visit (Vax 1)	1-Week Follow-up Visit (Vax 1)	1-Month Follow-up Visit	6-Month Follow-up Visit	Potential COVID-19 Illness Visit	Potential COVID-19 Convalescent Visit
Visit window (Days)	0 to 28 Days Before Visit 1	Day 1	1 to 3 Days After Visit 1	6 to 8 Days After Visit 1	28 to 35 Days After Visit 1	154 to 168 Days After Visit 1	Optimally Within 3 Days After Potential COVID-19 Illness Onset	28 to 35 Days After Potential COVID-19 Illness Visit
Physical examination (height, weight, BMI, vital signs, general appearance, examination of skin, auscultation of the heart, auscultation of the lungs, palpation of the abdomen, examination of extremities)	X	X	X	X	X	X		
ECG	X			X				
Collect blood sample for hematology and chemistry^a^	~13.5 ml	~13.5ml	~13.5 ml	~13.5 ml	~13.5 ml	~13.5 ml		
Collect screening blood sample for HIV, HBsAg, HBc Ab, and HCV Ab tests	X included in chemistry sample							
Perform alcohol test	X							
Perform drug panel test	X							
Perform serum pregnancy test (if appropriate)	X							
Perform urine pregnancy test (if appropriate)		X				X		
Urinalysis^a^	X	X				X		
Obtain nasal swab (mid-turbinate) for COVID-19	X	X^b^					X	
Collect non-study vaccine information	X	X	X	X	X	X		
Confirm eligibility	X	X						
Collect prohibited medication use	X	X	X	X	X	X	X	X
Review hematology and chemistry results^c^	X	X	X	X	X	X		
Review temporary delay criteria		X						
Confirm use of contraceptives (if applicable)	X	X	X	X	X			
Obtain randomization number and study intervention allocation		X						
Collect blood sample for immunogenicity assessment^a^		~20 ml			~20 ml	~20 ml (optional 50 mL^d)^		
Administer study intervention^c^		X						
Assess acute reactions after study intervention administration^c^		X						
Explain participant communication methods (including for diary completion)		X						
Provide thermometer measuring device		X						
Provide diary to subjects		X						
Review reactogenicity diary data			X	X	X	X		
Review ongoing reactogenicity diary symptoms and obtain stop dates			X	X				
Collect diary to assess participant				X				
Collect AEs and SAEs as appropriate	X	X	X	X	X	X	X	X
Collection of COVID-19-related clinical and laboratory information							X	X

HBc Ab, hepatitis B core antibody; HBsAg, hepatitis B surface antigen; HCV Ab, hepatitis C virus antibody; HIV, human immunodeficiency virus; min, minutes; NAAT, nucleic acid amplification test; vax, vaccination.

^a^Availability of results will not delay further study activities.

^b^Two swabs will be taken at 1–2 days prior to Visit 1. One will be tested (at the site, if possible, otherwise at the central laboratory) within 48 h, and vaccination will only proceed if it is NAAT-negative for SARS-CoV-2 genomes. The second will be sent to the central laboratory for potential later testing.

^c^The first two participants vaccinated in each cohort will be observed at the site for at least 4 h after study intervention administration, with each subject to be monitored for 60 min before proceeding to the next subject. The next four participants in each cohort may be vaccinated at least 48 h after the first two participants have completed their Visit 2 safety data review and completed 48 h post-vaccination, and they will also be observed for at least 4 h after study intervention administration at the study site. Vaccination of the rest of the subjects in each cohort will commence no sooner than 24 h after the sixth participant has received their vaccination. Post-immunization local and systemic AEs and vital sign measurements will be performed by site staff at hourly intervals. After 4 h post-vaccine administration through 7 days after vaccination, solicited local and systemic AEs and body temperature measurements will be reported daily by the study participant.

## Methods: participants, interventions, and outcomes

### Study setting {9}

Male and female participants between the ages of 18 and 55 years at the time of enrollment are eligible for the study. The participants should be healthy as determined by their medical history, physical examination, and clinical verification by the investigator. All participants should have received at least 2 doses of an approved mRNA COVID-19 vaccine at least 4 months prior to enrollment. Participants will be enrolled at the Clinical Trial Unit at King Abdullah International Medical Research Center (KAIMRC), Kingdom of Saudi Arabia.

### Eligibility criteria {10}

#### Inclusion criteria

Potential study participants who meet ALL the following inclusion criteria will be eligible to participate in the study:

1. Male or female individuals between the ages of 18 and 55 years (inclusive) at the time of enrollment.
2. Healthy individuals, as determined based on medical history, physical examination, and clinical verification by the investigator.
3. Individuals who agree to commit to complying with planned scheduled visits, vaccination, laboratory tests, and any other procedures.
4. Individuals who have received at least 2 doses of an approved mRNA COVID-19 vaccine at least 4 months prior to enrollment.
5. Female subjects of childbearing potential must have used a highly effective contraceptive method for at least 60 days prior to receiving the first dose of the investigational vaccine and should continue using contraception for at least 1 month after Visit 5 (end of study). Post-menopausal female subjects with at least 12 months of amenorrhea (without an alternative medical cause) can be enrolled.
6. Male subjects able to father children and sexually active with a female partner who is able to bear children must be willing to use (or have their female partner use) a highly effective contraceptive method from the time they receive the first dose of the study vaccine until at least 1 month after Visit 5.
7. Individuals must be willing to sign the informed consent form (ICF), which includes all the requirements and restrictions listed in the ICF and the study protocol.

#### Exclusion criteria

Potential study participants who meet ANY of the following exclusion criteria will not be eligible to participate in the study:

1. Individuals with known infection with Hepatitis B virus (HBV), hepatitis C virus (HCV), or human immunodeficiency virus (HIV).
2. Individuals currently infected and diagnosed with COVID-19 as documented by a PCR nasal swab test.
3. Individuals who have received an mRNA-based COVID-19 vaccine within 4 months of first study drug administration.
4. Individuals who have received only one dose of a COVID-19 vaccine.
5. Individuals working in a facility with a high probability of SARS-CoV-2 infection, such as health workers in hospitals.
6. Individuals with a history of severe allergic reactions and/or anaphylaxis and/or serious adverse reactions associated with vaccines and/or allergy or hypersensitivity to any component of the study intervention.
7. Individuals using immunosuppressive therapy.
8. Individuals receiving treatment or medications that can adversely affect the immune system in the 90 days before first investigational vaccine administration, including but not limited to: interferon, immunoglobulin, immunomodulators, epinephrine injectors, and cytotoxic drugs.
9. Individuals diagnosed with any diseases or conditions that are associated with severe COVID-19, including the following factors:
  - Diabetes Mellitus
  - Hypertension
  - Asthma
  - BMI more than 30 kg/m^2^
  - Chronic pulmonary disease
  - Chronic liver diseases
  - Chronic renal diseases
10. Individuals with known or suspected immunological disorders, including autoimmune disease and diabetes mellitus.
11. Individuals with current or previous neurological disorders, such as epilepsy or Guillain-Barré syndrome.
12. Individuals with psychiatric disorders or cognitive impairment.
13. Individuals with bleeding disorders or other conditions associated with prolonged bleeding time.
14. Individuals with clinically significant abnormal safety laboratory results at screening, in the opinion of the investigator (retesting is allowed).
15. Individuals receiving or planning to receive a non-study vaccine 30 days prior to study enrollment or during the study participation interval. Influenza vaccination is acceptable.
16. Individuals receiving blood or donating blood or blood components 60 days prior to study enrollment or planning to receive or donate blood or blood components during the study.
17. Individuals participating in a clinical trial (investigational vaccine, treatment, or device) 30 days prior to study enrollment or planning to participate in other clinical trials during the study.
18. Individuals intending to participate in another clinical trial while enrolled in this study.
19. Individuals with a positive alcohol or drug test or with a history of alcohol or drug addiction.
20. Individuals with other health conditions that may compromise their health and safety while participating in the clinical trial or that may compromise the study's primary or secondary objectives, in the opinion of the investigator.
21. Female participants who are pregnant, planning to become pregnant, or are breastfeeding.

### Who will take informed consent? {26}

Prior to screening, each subject shall submit a signed consent indicating their agreement to participate in the study. The subject will be given a designated screening number that will be documented in the screening and enrollment log. The principal investigator is responsible for ensuring that informed consent is given by all potential study participants.

## Intervention

### Intervention description {11a}

The drug product is prepared using an aseptic fill and finish process and supplied in glass vials as a liquid for injection, where each dose will be filled in an individual glass vial according to the pDNA concentration in the study (1 mg, 2 mg, and 4 mg). The sponsor will be responsible for ensuring the correct labeling of the study vaccine in accordance with the legal and regulatory compliance at the study site where this Phase 1 study will be performed. The packing of the clinical batch will be up to 50 vials per carton. The drug product batch is stored at optimal temperature (-20°C) until usage. The sponsor will ensure the supply of the candidate COVID-19 pDNA vaccine to the designated study site according to GCP and Good Distribution Practice (GDP). The temperature monitoring device will be included per shipping container.

The investigational product, a vaccine, should be received at the designated study site by the investigator/designee. The study investigator/designee must ensure the following:

- The vaccine is received at the appropriate temperature for shipment (-20°C) and verified by the downloaded data logger.
- The vaccine is received in good condition.
- The proper, temperature-appropriate storage of the vaccine in temperature-monitored freezers.
- The storage of the vaccine at a safe, secure, and locked location.
- Appropriate inventory and record documentation to ensure a proper storage temperature.

The storage of the investigational vaccine vials will be monitored by the study monitors during site visits. The unused investigational vaccine vials must be returned to the sponsor.

The investigational vaccine vials for this study will be stored at the appropriate temperature in a secure place under appropriate freezing conditions with temperature monitoring. The expiration date for the vaccine should be checked prior to vaccine administration to the subject. Expired vaccines MUST NOT be administered to the study subjects. If deviation occur the IMP must be immediately quarantine and labeled “Do Not USE”. The Sponsor should be notified within 24 h and the Sponsor shall review the temperature data and assess the impact of product quality. The IMP will either be released for use, destroyed, or returned to the Sponsor based on the investigation outcome.

Three dose levels of the COVID-19 pDNA vaccine vs. placebo will be tested in this study. Subjects will be randomized into three different dosing cohorts. In each cohort, 14 subjects will undergo the IM administration of vaccine or placebo (Saline) at a 3:1 allocation ratio. Subjects that are assigned to receive the vaccine will be administered at one of the following dose levels:

- Cohort 1: 1 mg
- Cohort 2: 2 mg
- Cohort 3: 4 mg

Participants will be receiving the investigational vaccine or placebo at Visit 1 only. The dose is described in the IB. The study intervention will be administered IM, preferably in the deltoid muscle of the non-dominant arm. The liquid for injection will be withdrawn from the ampoule into a conventional syringe with a needle appropriate for IM administration, as described in the pharmacy manual. Administration of the vaccine should be performed by a qualified, GCP-trained member of study staff in accordance with the institutional and country-specific guidance. Standard vaccination practice should be observed and monitored.

### Criteria for discounting allocated intervention {11b}

When a subject withdraws their consent, they can no longer participate in this study. No new study data will be collected, but the study data that have already been collected will continue to be used and processed to maintain the integrity of the research in accordance with applicable data protection law (Guidelines 07/2020 on the concepts of controller and processor in the GDPR, Version 1.0, 02 September 2020). If a subject withdraws from the study, all study data compiled to date will be used in the study. No additional study data will be compiled, except for follow-up activities, and the subject may request that retained identifiable samples be destroyed to prevent further analysis. The site may contact the withdrawn subject to obtain health information for an Early Termination Visit. Subjects will not be identified in any report or publication.

#### Early termination visit

If a subject withdraws from the study, the following steps should be followed:

- Receipt of diary cards
- Review of the safety data
- Review of concomitant medications and vaccination received
- Evaluation of a subject's vital signs
- Confirmation of the use of contraceptives through at least 1 month after the end of study (Visit 5).
- All safety assessments applicable to Visit 5.

#### Involuntary withdrawal from the Study

The following are examples of reasons for the involuntary withdrawal of subjects from the study:

- Adverse events
- Subject fail to follow up
- Deviation in the protocol
- Death

### Strategies to improve adherence to interventions {11c}

The COVID-19 pDNA vaccine booster dose will be given once only. Therefore, patient adherence to the intervention is not applicable.

### Prior and concomitant medications {11d}

All prior and concomitant medications, vaccines, and blood products will be collected in the electronic case record form (eCRF). Concomitant therapy or treatment associated with SAEs should be collected in the eCRF from signing of the ICF until the end of the study. In addition, concomitant therapy associated with unsolicited AEs occurring from the time of day of vaccination (Visit1) until 21 days, should be collected and recorded in the eCRF. Concomitant medications associated with solicited AEs occurring from the time of vaccination until 7 days after vaccination should also be collected and recorded in the eCRF.

A subject receiving any of the following medications will be excluded from the study:

- Statin medications up to 30 days of enrollment through the end of the study.
- Corticosteroids up to 30 days of enrollment. Inhaled and topical corticosteroids are permitted.
- Antimetabolite or alkylating drugs up to 30 days of enrollment through the end of the study.
- Treatments or medications that can adversely affect the immune system, such as interferon, immunoglobulin, immunomodulators, epinephrine injectors, cytotoxic drugs, and immune-modulating drugs, within 90 days of enrollment.
- Radiation therapy within 60 days of enrollment until the end of the study.
- Donating or receiving blood or plasma products up to 60 days of enrollment or planning to receive or donate blood and blood components throughout the study.
- Non-study vaccines up to 30 days prior to study enrollment until end of study with the exception of an influenza vaccine.

If a subject requires any treatment listed above or in the exclusion criteria, discontinuation of the subject from the study should be considered. The use of nutritional/herbal supplements or other medications such as antihistaminic drugs during screening and throughout the study period is not permitted. Subjects are permitted to use antipyretic drugs, such as paracetamol (up to a 1 g dose) for the treatment of any local or systemic reactions that can be associated with the study drug. Alternatively, a 500 mg dose of paracetamol can be taken every 6 h up to 36 h.

### Outcomes {12}

The study outcomes include safety and immunogenicity endpoints and are summarized in Table 2.

#### Primary endpoint (safety and tolerability)

The primary endpoint includes assessments of the safety and tolerability of the investigational pDNA COVID-19 vaccine in healthy adults administered as a single dose vs. placebo.

These assessments include four subcategories:

- Local reactions (i.e., reactogenicity) for up to 7 days following vaccination
- Systemic events for up to 7 days following vaccination
- Adverse events (AEs) for up to 1 month following vaccination
- Serious AEs (SAEs) for up to 6 months following vaccination

Recording of unsolicited AEs and SAEs—reactogenicity to the vaccine will be assessed based on the percentage and frequency of AEs up to 7 days after receiving a study vaccine dose. The percentage of subjects experiencing unsolicited AEs up to 30 days after receiving a study vaccine will also be determined.

#### Secondary endpoints (Immunogenicity)

1. Assessment of bAB and nAB titers as indicators of the humoral immune response after vaccination compared to placebo.
2. Assessment of T cell and intracellular cytokine responses as indicators of the cell-mediated immune response after vaccination compared to placebo.

#### Exploratory endpoint

In addition, the immunogenicity endpoint includes further exploration of immunogenicity after vaccination vs. placebo by evaluating antibody responses for SARS-CoV-2 variants of concern.

### Participants timelines {13}

The participants' SOAs and timeline are shown in Table 3.

### Sample size {14}

Laboratory analyses will include safety assessments at a local laboratory and immunogenicity assessment at a central laboratory. In total, approximately 276 ml of blood will be collected throughout all study visits for safety and immunogenicity assessment. Approximately 13.5 ml of blood will be withdrawn from each study subject at the following screening visits: Visit 1, Visit 2, Visit 3, Visit 4, and Visit 5 (all study visits). A total amount of approximately 67.5 ml of blood will be collected for safety assessment throughout the study. An overall volume of approximately 20 ml of blood will be withdrawn from each study subject, collected during the entire study period for immunogenicity assessment during Visit 1, Visit 4, and Visit 5. A total amount of 100 ml of blood will be collected for immunogenicity assessment throughout the study. Samples will be tested at a central laboratory for the assessment of bAb, nAb, and cellular immunity.

### Recruitment {15}

Recruitment will occur in 4–6 weeks with 8 months of follow-up.

## Assignment of intervention: allocation

### Randomization {16}

When an individual is determined to be eligible to participate in the study, the subject will be enrolled in a computerized randomization system that assigns a designated subject ID to allocate them to receive either the COVID-19 pDNA vaccine or placebo using Interactive Response Technology (IRT). Each cohort will contain 14 subjects. At Visit 1, the first two subjects of each cohort will be randomized according to a separate randomization list. One subject will be randomized to placebo, and one subject will be randomized to the study treatment to ensure that at least one subject will receive the study vaccine. They will be monitored for 4 h, with each subject to be monitored for 60 min before proceeding to the next subject. The next four participants in each cohort will be vaccinated at least 48 h after the first 2 participants complete their visit 2 safety data review. They will also be observed for at least 4 h at the study site. Vaccination of the rest of the subjects in each cohort will commence no sooner than 24 h after the sixth participant has undergone vaccination. All subjects in the dosing visit (Visit 1) will be observed for 4 h.

### Concealment mechanism {16b}

Randomization will be performed using a web-based IRT randomization system hosted by Icon plc. Concealment will be maintained until the registration and randomization process has been completed.

### Implementation {16c}

The study team from Icon plc will be in charge of the randomization using the web-based randomization system.

## Assignment of intervention: blinding

### Who will be blinded {17a}

This is a double-blinded study. The sponsor, investigators, subjects, monitors, site personnel, and laboratory staff will be blinded to the study treatment given with the exception of unblinded pharmacist (s) and an unblinded biostatistician. Unblinded pharmacists who will be assigned to dispense the study treatement or the placebo and unblinded nurses will be assigned to adminsiter the study treatment or placebo in the pharmacy. The vaccine syringes should be labeled in a manner to preserve blinding.

In addition, an independent unblinded data monitoring committee (DMC) biostatistician will be assigned to review the safety data. This biostatistician will be independent of all staff involved in the evaluation of the study data reviewed by the DMC. The DMC members will receive unblinded safety data to decide on dose escalation for the next cohort. Interim safety analysis will be conducted by an independent unblinded data monitoring committee (DMC) biostatistician for all visits until Visit 5. Interim safety results will be confirmed by the DMC in accordance with the DMC charter.

### Procedure for unblinding if needed {17b}

In cases of urgent medical conditions and in emergency situations, individual unblinding can be performed for the reasons of subject safety and when knowledge of the investigational medical product (active or placebo) is essential for the clinical management and wellbeing of the subject. In this case, the investigator will retrieve the subject's assigned vaccine from the computerized randomization system by IRT.

## Data collection and management

### Plan for assessment and collection of outcomes {18a}

Safety assessments will be performed by measuring the local and systemic solicited AEs, unsolicited AEs, SAEs, as well as safety laboratory data for the study subjects. In addition, tolerability will be assessed for the vaccine. Tolerability will be assessed by the dual (joint) evaluation of both reactogenicity and unsolicited AEs. The time point for all safety assessments is described in the SOA. For each cohort, the first two subjects will receive the study vaccine and will be monitored for adverse reactions (AEs or SAEs) within the first 4 h. The next four participants in each cohort may be vaccinated at least 48 h after the first two participants have completed their Visit 2 safety data review and completed 48 h post-vaccination and they will also be observed for at least 4 h after study intervention administration at the study site. Vaccination of the rest of the subjects in each cohort will commence no sooner than 24 h after the sixth participant has received their vaccination, after which the next four subjects will receive the study vaccine and will be monitored for 4 h. Then, 24 h following vaccination, the remainder of the study cohort will receive the vaccine and will be monitored for 4 h. All subjects receiving their study vaccine dose will be observed for 4 h. All assessments will be documented in the eCRF as well as the AE and SAE forms. Further, safety parameters included in the diary card for will be collected from each participant. The types of the symptoms, severity, and the treatments to reduce symptoms at home will be recorded the diary card by the participant. The diary card are collected and assessed by the physician at each visit as described in the SOA (Table 3).

Assessment of reactogenicity will be performed by measuring the percentage and frequency of subjects with solicited local and solicited systemic AEs up to 7 days after each vaccination. Assessment of AEs will be performed by measuring the percentage and frequency of subjects with unsolicited AEs for 30 days after vaccination. For all cohorts, AEs will be monitored for 30 days after Visit 1. Assessment of SAEs will be performed by measuring the percentage and the frequency of subjects with SAEs from the start of the study until completion.

### Plans to promote participant retention and complete follow-up {18b}

The study team will provide reminders to study subjects prior to the vaccination visit and their follow-up visits. Study subjects will be reimbursed regularly for their time and transportation costs at every study visit.

### Data management {19}

Data for each study applicant should be reported in a case record form (CRF). All study data will be recorded and stored in an electronic format (eCRF). All data entries are checked and monitored by the sponsor and the study site. Clinical study data, including safety data as well as laboratory data and immunogenicity data, should be written in a source document and recorded in an eCRF. Safety data will include physical examination, medical history, AEs, SAEs, reactogenicity data, and diary information. The Investigator is responsible for verifying that data entries for all study participants are accurate and correct by physically or electronically signing the eCRF.

The data will be collected in a secure system and saved using an electronic data capture (EDC) system that enables password protection. The eCRF is integrated into the Rave, Medidata EDC system through a secure website that utilizes cloud access for enhanced efficiency and controlled accessibility of the clinical study. The Sponsor is responsible for the data management of this study, including quality checks of the data as well as data protection.

This study will be conducted in compliance with ICH CGP guidelines and in accordance with the most recent version of the Declaration of Helsinki. Records and documents, including signed ICFs, pertaining to the conduct of this study must be retained by the Investigator for 25 years after study completion unless local regulations or institutional policies require a longer retention period. The Sponsor, which is designated as the data controller, will confirm compliance of the GDPR Regulation (EU) 2016/679 throughout all stages of study data management.

### Confidentiality {27}

All subject data, clinical documents, and study findings will be considered confidential. The study site will collect, process, and store subject data according to the site's data protection and privacy policy and in compliance with applicable laws and regulations governing the processing of personal data. The Sponsor, investigator, and other study personnel must not disclose information prior to written approval from the Chief Principal Investigator.

### Plans for collection, laboratory evaluation, and storage of biological specimens for immunogenicity analysis {33}

Humoral and cellular immune responses elicited after COVID-19 vaccine candidate administration will be evaluated. Approximately twenty (19) ml of blood will be taken from each subject during Visit 1, Visit 4, and Visit 5. A total of 60 ml of blood will be collected for immunogenicity assessment throughout the study. Blood must be drawn prior to administration of the investigational vaccine. An optional blood draw of −50 ml will be taken at Visit 5 (from selected participants who consent) for exploratory COVID-19 research. All immunogenicity testing will be performed at accredited central laboratories and by qualified staff.

### Immunogenicity assessment

#### Humoral immune response

Approximately 4 ml of whole blood will be taken during Visit 1, Visit 4, and Visit 5. The blood must be drawn prior to vaccination of the subjects. The serum should be separated from the whole blood prior to conducting humoral antibody response assessments.

The humoral immune response will be evaluated as follows:

- Binding antibody responses to the S.FL, S1, and RBD S1 of wild-type SARS-CoV-2 using ELISA.
- Binding antibody response to the S.FL of SARS-CoV-2 variants of concern using V-plex COVID-19 panel.
- Neutralizing antibody directed against wild-type SARS-CoV-2 using V-plex assay COVID-19 (ACE2) neutralizing assay kit.
- Neutralizing antibody directed against SARS-CoV-2 variants of concern using V-plex COVID-19 panel.

Antibody response to the spike protein of SARS-CoV-2 will be evaluated for all subjects by measurement of the bAB and nAB responses as follows:

##### Binding antibodies

Assessment of bAB responses directed against S.FL, S1, and RBD of wild-type SARS-CoV-2 at Visit 1, Visit 4, and Visit 5.

Assessment of bAB responses directed against S.FL of SARS-CoV-2 variants of concern at Visit 1, Visit 4, and Visit 5.

##### Neutralizing antibodies

Assessment of nAB responses against wild-type SARS-CoV-2 at Visit 1, Visit 4, and Visit 5.

Assessment of nAB responses against SARS-CoV-2 variants of concern at Visit 1, Visit 4, and Visit 5.

#### Cellular immunity assessment

Cellular immunity will be evaluated in all recruited subjects. Approximately 16 ml of blood will be taken from each subject during Visit 1, Visit 4, and Visit 5. The blood should be drawn prior to vaccination of the subjects. Peripheral blood mononuclear cells (PBMCs) will be collected for the assessment of cell-mediated immunity using intracellular cytokines staining.

## Statistical methods

### Statistical methods for primary and secondary outcomes {20a}

#### Safety and tolerability endpoints and analysis

Safety and tolerability data will be evaluated using descriptive summary statistics for local reactions, systemic events, AEs/SAEs, and abnormal hematology and chemistry laboratory parameters, for each vaccine cohort. The sympotoms and corresponding grades for local AE, SAE, and other adverse events are categorized based on ICH E2A definitions and NCI Common Terminology Criteria for Adverse Events (CTCAE) and are summerized in Tables 4–6, respectievely.

**Table 4:** Local adverse events at administration site (i.e., reactogenicity).

Symptom/Sign	Grade 1	Grade 2	Grade 3	Grade 4
Pain	Pain or tenderness causing no or minimal limitation of use of limb	Pain or tenderness causing greater than minimal limitation of use of limb	Pain or tenderness causing inability to perform usual social & functional activities	Pain or tenderness causing inability to perform basic self–care function OR Hospitalization indicated
Rash/Redness	2.5 to < 5 cm in diameter OR 6.25 to < 25 cm^2^ surface area AND Symptoms causing no or minimal interference with usual social & functional activities	≥5 to < 10 cm in diameter OR ≥ 25 to < 100 cm^2^ area OR Symptoms causing greater than minimal interference with usual social & functional activities	≥10 cm in diameter OR ≥100 cm^2^ surface area OR Ulceration OR Secondary infection OR Phlebitis OR Sterile abscess OR Drainage OR Symptoms causing inability to perform usual social & functional activities	Potentially life– threatening consequences (e.g., abscess, exfoliative dermatitis, necrosis involving dermis or deeper tissue)
Induration/Swelling	2.5 to < 5 cm in diameter or 6.25 to < 25 cm^2^ surface area AND Symptoms causing no or minimal interference with usual social & functional activities	≥5 to < 10 cm in diameter OR ≥25 to < 100 cm^2^ area OR Symptoms causing greater than minimal interference with usual social & functional	≥10 cm in diameter OR ≥10 cm^2^ surface area OR Ulceration OR Secondary infection OR Phlebitis OR Sterile abscess OR Drainage OR Symptoms causing inability to perform usual social & functional activities	Potentially life–threatening consequences (e.g., abscess, exfoliative dermatitis, necrosis involving dermis or deeper tissue)

**Table 5:** Adverse events at non–injection site (systemic).

Symptom/Sign	Grade 1	Grade 2	Grade 3	Grade 4
Vomiting	Transient or intermittent AND No or minimal interference with oral intake	Frequent episodes with no or mild dehydration	Persistent vomiting resulting in orthostatic hypotension OR Aggressive	Life–threatening consequences (e.g., hypotensive shock)
Diarrhea	Transient or intermittent episodes of unformed stools OR Increase of ≤ 3 stools over baseline per 24–h period	Persistent episodes of unformed to watery stools OR Increase of 4 to 6 stools over baseline per 24–h period	Increase of ≥7 stools per 24–h period OR IV fluid replacement indicated	Life–threatening consequences (e.g., hypotensive shock)
Fever (temperature in °C)	38.0– < 38.6	38.6– < 39.3	39.3– < 40.0	≥40.0
Nausea	Transient (< 24 h) or intermittent AND No or minimal interference with oral intake	Persistent nausea resulting in decreased oral intake for 24 to 48 h	Persistent nausea resulting in minimal oral intake for > 48 h OR Rehydration indicated (e.g., IV fluids)	Life–threatening consequences (e.g., hypotensive shock)
Abnormal skin mucous membranes	Erythema, pruritus, color change	Diffuse rash, maculopapular rash, dry, desquamation	Bleb, effusion, desquamation, ulceration	Exfoliative dermatitis involves mucous, or polymorphic erythema, or is suspected of Stevens–Johnsons syndrome
Acute allergic reaction	Localized urticaria (wheals) with no medical intervention indicated	Localized urticaria with intervention indicated OR Mild angioedema with no intervention indicated	Generalized urticaria OR Angioedema with intervention indicated OR Symptoms of mild bronchospasm	Acute anaphylaxis OR Life–threatening bronchospasm OR Laryngeal edema
Fatigue, tiredness	Symptoms causing no or minimal interference with usual social & functional activities	Symptoms causing greater than minimal interference with usual social & functional activities	Symptoms causing inability to perform usual social & functional activities	Incapacitating symptoms of fatigue or malaise causing inability to perform basic self–care functions

**Table 6:** Other adverse events—general principles for classification of adverse events.

Grade 1	Grade 2	Grade 3	Grade 4	Grade 5
Mild: short duration (< 48h) or mild discomfort, causing no interference with daily life with no medical intervention indicated	Moderate: causing mild or moderate limitation of use of limb, with no or mild medical intervention indicated	Severe: causing greater than minimal limitation of use of limb, with medical intervention or hospitalization indicated	Critical: may be life–threatening, causing severe limitation of use of limb, with intensive hospitalization indicated	Death

The safety and toelrabilty analyses will be evaluated as follows:

- Assessment of administration site reactions (local reactions, i.e., reactogenicity)—frequency of local solicited AEs
- Assessment of systemic AEs—frequency of systemic solicited AEs
- Clinical assessment of participants' general health status
- Clinical laboratory investigations [hematology, clinical chemistry, urinalysis, and pregnancy tests (female participants)]—percentage of participants with clinically significant abnormal hematology and chemistry laboratory values at 1 and 7 days after Visit 1; and 30 days after Visit 1; percentage of participants with graded shifts in hematology and chemistry laboratory assessments between Visit 1 and 1 and 7 days after Visit 1; and 30 days after Visit 1.
- Monitoring of participants' COVID-19 infection status.
- Recording of unsolicited AEs and SAEs—reactogenicity to the vaccine will be assessed based on the percentage and frequency of AEs up to 7 days after receiving a study vaccine dose. The percentage of subjects experiencing unsolicited AEs up to 30 days after receiving a study vaccine will also be determined.
- Recording of concomitant medication use
- AEs and SAEs will be collected from the first administration of the investigational vaccine until study completion.

### Immunogenicity endpoints and analysis

Immunogenicity evaluation will be conducted as follows:

- Evaluation of bAB antibody responses specific for wild-type SARS-CoV-2—GMT of serum anti-S.FL, anti-S1, and anti-RBD bAB at Visit 1 (baseline) and Visit 4 (30 days after Visit 1).
- Evaluation of nAB antibody responses specific for wild-type SARS-CoV-2—GMT of serum anti-S nAB at Visit 1 (baseline) and Visit 4 (30 days after Visit 1).
- Evaluation of the bAB and nAB ratio for wild-type SARS-CoV-2—the ratio of bAB and nAB titers directed against the investigational pDNA vaccine at Visit 1 (baseline) and Visit 4 (30 days after Visit 1).
- Evaluation of the fold rise in bAB responses for wild-type SARS-CoV-2—GMFR and proportion of participants with a 4-fold increase in serum anti-S.FL, anti-S1, and anti-RBD binding antibody titers at Visit 1 (baseline) and Visit 4 (30 days after Visit 1).
- Evaluation of cellular immune responses (CD3, CD4, CD8, CD45RA, CCR7, IFN-γ, IL-2, IL-4, IL-13, and TNF) at Visit 1 (baseline) and Visit 4 (30 days after Visit 1).

#### Binding antibody evaluation

- Values below the detection limit (1:50).
- Antibody measurements will be logarithmically calculated (log 10)

#### Neutralizing antibody evaluation

- Values below the detection limit (1:20)
- Antibody measurement will be logarithmically calculated (log 10)

### Exploratory immunogenicity endpoints and analysis (exploratory analysis)

- Evaluation of bAB antibody responses for SARS-CoV-2 variants of concern—GMT of serum anti-S bAB at Visit 1 (baseline) and Visit 4 (30 days after Visit 1).
- Evaluation of nAB antibody responses for SARS-CoV-2 variants of concern—GMT of the serum anti-S nAB at Visit 1 (baseline) and Visit 4 (30 days after Visit 1).
- Evaluation of the bAB and nAB ratio for SARS-CoV-2 variants of concern—the ratio of bAB and nAB titers directed against the investigational pDNA vaccine at Visit 1 (baseline) and Visit 4 (30 days after Visit 1).

### Interim analysis {21}

An interim analysis of immunogenicity and safety data will be conducted after completion of safety and immunogenicity assays for all the samples through Visit 4. The analysis will include safety data for all subjects at each visit until Visit 4. Interim immunogenicity analysis will include immunogenicity data from all subjects at Visit 1 and Visit 4. This interim data will be used to evaluate the optimal dosage for use in future clinical trials.

## Oversight and monitoring

### Composition of data monitoring committee {5d}

A DMC by Icon plc will be implemented for this Phase 1 COVID-19 vaccine trial for the purpose of reviewing the safety data of participants during their periodic visits. The role of the DMC is to review safety data as described in the DMC charter. Escalation between doses will be based on DMC review. Dose escalation will start at least 12–14 days after the previous cohort received the study vaccine for (Cohort 1 and Cohort 2) and (Cohort 2 and Cohort 3).

### Adverse events reporting and harm {22}

All AEs, SAEs, and Serious Adverse Reactions (SARs) will be monitored in each participant after the administration of the investigational vaccine. All SAEs and AEs should be documented in the eCRF as well as the AE and SAE forms. In addition, any medications and therapeutics used should be reported in the eCRF. The investigator must notify the sponsor and the DMC of AEs and SAEs. The investigator will immediately notify the sponsor (within 24 h) of any SAE to comply with any ethical and/or legal obligations toward the study participants and to ensure that all stopping/halting rules are met. The sponsor will inform the DMC. If any halting rules are to be applied, vaccination of the subjects must be stopped until a full review of safety data by the DMC and health authorities has been performed. For all subjects who experience a SAE, follow-up information deemed as significantly new by the investigator will be reported no later than 24 h after they have been made aware, and follow-up information deemed as not significantly new by the investigator will be reported no later than the next business day after they have been made aware.

#### Stopping rules

The stopping rules will be applied based on AEs, SAEs, clinical laboratory assessments, and diary card data. These data will be monitored by the investigator and sponsor for the purpose of identifying possible risk.

The following are considered as stopping/pausing rules in this Phase 1 study:

- A participant receiving the investigational vaccine (in any cohort) who develops Grade 4 AEs or laboratory abnormalities that are assessed by the investigator as possibly related to the vaccine with no other alternative or plausible causes. Vaccination will be stopped until a full safety review is completed.
- Two or more participants receiving the investigational vaccine (in any cohort) who develop Grade 3 AEs or laboratory abnormalities after vaccination that are assessed by the investigator as possibly related to the vaccine with no other alternative or plausible causes. Vaccination will be stopped until a full safety review is completed.
- A participant receiving the investigational vaccine (in any cohort) develops a fever (>40°C) for more than 24 h after receiving the vaccine with no other alternative or plausible causes. Vaccination will be stopped until a full safety review is completed.
- If a participant in any cohort requires admission to an intensive care unit (ICU) for COVID−19 infection after receiving the investigational vaccine that is assessed as possibly related to the vaccine with no other plausible or attributable causes. Vaccination will be stopped until a full safety review is completed.
- A participant receiving the investigational vaccine (in any cohort) who develops a SAE that is assessed by the investigator as possibly related to the vaccine with no other alternative or plausible causes. Vaccination will be stopped until a full safety review is completed.
- A participant receiving the investigational vaccine (in any cohort) who develops a serious adverse reaction that is assessed by the investigator as possibly related to the vaccine with no other alternative or plausible causes. Vaccination will be stopped until a full safety review is completed.
- Two or more participants receiving the investigational vaccine (in the same cohort) who develop severe non-serious adverse reactions, whether or not they are within the same organ class.
- If a participant in any cohort dies and this is aassessed by the investigator as possibly related to the investigational vaccine. In this case, all vaccination will be halted.

### Frequency and plan for inspecting trial conduct {23}

Quality assurance (QA) includes all the planned and systematic actions that are established to ensure that the clinical study is performed, and the data are generated, documented (recorded), and reported according to ICH GCP and local/regional regulatory standards. An inspection from the Sponsor or qualified designee, who is independent of and separated from routine monitoring, may periodically arrange inspections/audits of the clinical study by reviewing the data obtained and procedural aspects. These inspections may include on-site inspections/audits and source data checks. Direct access to source documents is required for the purpose of these periodic inspections/audits. This study will be conducted in compliance with ICH CGP guidelines. This study will also be conducted in accordance with the most recent version of the Declaration of Helsinki. Records and documents, including signed ICFs, pertaining to the conduct of this study must be retained by the Investigator for 25 years after study completion unless local regulations or institutional policies require a longer retention period. No records may be destroyed during the retention period without the written approval from the Sponsor. No records may be transferred to another location or party without written notification of the Sponsor.

### Plans for communicating important protocol amendments to relevant parties (e.g., trial participants, ethical committees) {25}

Study procedures will not be changed without the mutual agreement of the PI and the sponsor. If there are any changes to the study protocol, these changes will be documented in a study protocol amendment. If a protocol amendment requires a change to the ICF, the IRB Comitee must approve the revised ICF before it is used.

### Dissemination plans {31a}

Upon study completion, results will be published in a peer-reviewed medical scientific journal. Interim results may be released by the sponsor.

## Discussion

Current approved Covid-19 vaccines primarily utilize either mRNA technology or recombinant protein-based approaches. While highly effective in pandemic responses, each platform has distinct limitations. As single stranded molecule highly susceptible to enzymatic degradation, mRNA requires further sophisticated encapsulation with lipid nanoparticles for protection and efficient cellular delivery. Furthermore, this fragility necessitates an ultra cold chain shipment and storage (-70 °C) to prevent degradation. Conversely, protein based vaccines are generally more and easier to store but primarily induce humoral (antibody) responses. They are notably less efficient at activating cytotoxic T cells, which are crucial for destroying virus infected cells. In contrast, pDNA based vaccine platform offer unique and advantageous profile that addresses many of the issues. pDNA is stable, double stranded, circular molecule that can be store at refrigerated temperature or even with ambient temperature, which greatly reduce the costs associated with cold chain logistics. Once delivered into host cell, pDNA activates both arms of adaptive immune systems; the humoral and cellular responses. Furthermore, the pDNA vaccine include selfaduvinating activity that provoke innate immune responses (12.13). This balanced immune activation makes pDNA a promising platform for future vaccine strategies. As such, IAU has developed a thermally stable pDNA-based vaccine candidate using a platform approach that enables the rapid development of vaccines against emerging viral diseases, including SARS-CoV-2. The pDNA vaccine developed by IAU is a synthetic, codon-optimized sequence encoding either the full-length Spike (S.opt.FL) as genes of interest. Potential key advantages of this pDNA vaccine include that it is cost-effective, thermally stable, and can be used for multiple rounds of immunization without the limitations of anti-vector responses, making it an appealing choice as a booster dose. Given concern surrounding the continuous emergence of new COVID-19 variant strains that evade the immunity gained from previous rounds of vaccination, it will be essential to implement a vaccine platform that can be quickly adapted to newly emergent strains and on a regular/seasonal basis. The manufacturing of pDNA vaccines is robust, scalable, and highly adaptable. Moreover, pDNA manufacturing is conducted through a series of quick manufacturing steps, offering a unique advantage compared to the manufacturing of protein vaccines and even mRNA vaccines.

The IAU COVID-19 pDNA vaccine is formulated without the need for encapsulation using nanoparticles or the use of adjuvants. In addition, the codon-optimized S gene is inserted into the pVax1 plasmid backbone that is FDA-approved for clinical trials and has been tested in numerous preclinical and clinical studies. Importantly, unlike with the previously developed Covid-19 pDNA vaccines, the IAU pDNA is admistred without the application of physical delivery methods. In our previous preclinical studies, we demonstrated that the administration of S.opt.FL pDNA induces high levels of S-specific bAB and nAB against SARS-CoV-2. Notably, the pDNA vaccination predominantly elicited Th1-mediated immune responses, characterized by S-specific IgG2 and IFN-γ production. This Th1-skewed immune profile is essential in conferring protection against virus challenge (22, 23).

This study aims to investigate the safety, tolerability, and immunogenicity of this prophylactic vaccine against COVID-19 when administered as a booster heterologous vaccine in participants already receiving mRNA vaccine using a single ascending dose design. Previous clinical trial on Coronavirus pDNA vaccine found that increasing the doses to 6 mg did not yield superior immunogenicity, supporting the use of lower doses (28). Saline was selected as a control to provide a strictly inert baseline, ensuring a clear assessment of the vaccine's overall clinical impact in accordance with the internationally accepted safety standards. The study will offer valuable insight regarding the safety and immunogenicity of this pDNA vaccine as a booster vaccine in participants who have previously received a COVID-19 mRNA vaccine. In addition, this study will determine the safest maximum tolerated dose in participants receiving this pDNA vaccine. The study will also provide information regarding the humoral and cellular immune responses elicited by the vaccine and their durability in pDNA vaccine recipients. Lastly, as with other RNA viruses, it will be essential to explore the cross-protection of this SARS-CoV-2 vaccine against antigenically divergent strains (29–35), and this study will evaluate these effects through its exploratory objective. All of this data will be critical to determine which cohort to inform subsequent clinical trials.
